# Characterization and Pathogenicity of the Porcine Deltacoronavirus Isolated in Southwest China

**DOI:** 10.3390/v11111074

**Published:** 2019-11-18

**Authors:** Yujia Zhao, Huan Qu, Jingfei Hu, Jiayu Fu, Rui Chen, Cheng Li, Sanjie Cao, Yiping Wen, Rui Wu, Qin Zhao, Qigui Yan, Xintian Wen, Xiaobo Huang

**Affiliations:** 1 Research Center of Swine Disease, College of Veterinary Medicine, Sichuan Agricultural University, Chengdu 611130, China; zhaoyujia2015@163.com (Y.Z.); 18102341339@163.com (R.C.); csanjie@sicau.edu.cn (S.C.); yueliang5189@163.com (Y.W.); wurui1977@163.com (R.W.); yanqigui@126.com (Q.Y.); xintian3211@126.com (X.W.); 2 Sichuan Science-observation Experimental station of Veterinary Drugs and Veterinary Diagnostic Technology, Ministry of Agriculture, Chengdu 611130, China; 3 National Teaching and Experiment Center of Animal, Sichuan Agricultural University, Chengdu 611130, China

**Keywords:** porcine deltacoronavirus, isolates, phylogenetic analysis, pathogenicity

## Abstract

Porcine deltacoronavirus (PDCoV) is a newly emerging enteric pathogen in swine that causes diarrhea in neonatal piglets and creates an additional economic burden on porcine industries in Asia and North America. In this study, a PDCoV isolate, CHN-SC2015, was isolated from Sichuan Province in southwest China. The isolate was characterized by a cytopathic effect, immunofluorescence, and electron microscopy. CHN-SC2015 titers in LLC-PK cells ranged from 10^4.31^ to 10^8.22^ TCID_50_/mL during the first 30 passages. During serial passage, 11 nucleotide mutations occurred in the S gene, resulting in nine amino acid changes. A whole genome sequencing analysis demonstrated that CHN-SC2015 shares 97.5%–99.1% identity with 59 reference strains in GenBank. Furthermore, CHN-SC2015 contained 6-nt deletion and 9-nt insertion in the ORF1ab gene, 3-nt deletion in the S gene and 11-nt deletion in its 3′UTR compared with other reference strains available in GenBank. A phylogenetic analysis showed that CHN-SC2015 is more closely related to other PDCoV strains in China than to the strains from Southeast Asia, USA, Japan, and South Korea, indicating the diversity of genetic relationships and regional and epidemic characteristics among these strains. A recombination analysis indicated that CHN-SC2015 experienced recombination events between SHJS/SL/2016 and TT-1115. In vivo infection demonstrated that CHN-SC2015 is highly pathogenic to sucking piglets, causing diarrhea, vomiting, dehydration, and death. Virus was shed daily in the feces of infected piglets and upon necropsy, was found distributed in the gastrointestinal tract and in multiple organs. CHN-SC2015 is the first systematically characterized strain from southwest China hitherto reported. Our results enrich the body of information on the epidemiology, pathogenicity and molecular evolution associated with PDCoV.

## 1. Introduction

Porcine deltacoronavirus (PDCoV), belongs to the genus *Deltacoronavirus* within the family *Coronaviridae* [1]. It is an emerging swine enteric virus that causes diarrhea, vomiting, dehydration, and death in nursing piglets and the mortality rates are about 40%–80% [2]. The earliest identification and report of PDCoV was in Hong Kong in 2012 by Woo et al. [3] but it only began to receive much attention after an outbreak in the United States in 2014 [4]. Subsequently, it spread quickly through much of the United States [5], Korea [6], Canada [7], Japan [8], Vietnam [9] and Thailand [10], incurring enormous economic losses to the pork industry. In China, the prevalence of PDCoV was about 36.43% in suckling piglets in Henan province and 21.7% in Guangdong province. Moreover, co-infection with porcine epidemic diarrhea virus (PEDV) was common in infected piglets [11,12]. The first Chinese PDCoV strain, CHN-HN-2014, was reported in 2014 and was found to be closely related to the PDCoV strain HKU-155 [13]. Since then, several other PDCoV isolates have been reported in the pork producing provinces of China [12,14]. To date, complete genome sequence of known PDCoV strains are relatively conserved and share 97.1%–99.9% nucleotide identity [15]. Phylogenetic analyses based on the complete genome have suggested that PDCoV may have originated from a sparrow coronavirus [16]. In addition, Lau et al. found that the novel avian deltacoronavirus QuaCoV UAE-HKU30 from quails belonged to the same coronavirus species as porcine coronavirus HKU15 and sparrow coronavirus HKU17, providing an example of avian-to-swine transmission [17].

PDCoV is an enveloped positive-sense single-stranded RNA virus with a genome about 25.4 kb, which is the shortest genome among the known coronavirus [18]. The two opening reading frames (ORFs), ORF1a and ORF1ab, occupy almost two-thirds of its genome and encode two polymerase proteins, pp1a and pp1ab, respectively, which are proteolytically cleaved into 15 mature non-structural proteins [19]. The other one-third of the genome encodes four structural proteins: spike (S protein), envelope (E protein), membrane (M protein), and nucleoprotein (N protein) [20]. E and M are the transmembrane proteins and are involved in viral replication [21,22]. The N protein is highly conserved and plays a vital role in binding viral RNA [23]. Three accessory proteins, NS6 (located between M and N genes) and NS7/NS7a (located within N gene), are also found in the PDCoV genome [24,25]. These proteins may be associated with immune modulation and viral pathogenesis, although they are not essential for viral replication [26]. The E, M and N proteins may have potential to serve as efficient tools for the development of diagnostic assays and/or vaccines against PDCoV.

The PDCoV S protein interacts with host cell receptors and mediates the fusion of virus envelope to the host cytomembrane, [27] steps critical for viral entry. Many studies have demonstrated that mutations in the S protein affect the virus’ tropism and pathogenesis. For example, deletions in the S gene of porcine respiratory coronavirus (PRCV), a non-enteric pathogen derived from the transmissible gastroenteritis coronavirus (TGEV), alter its tropism and pathogenicity [28]. Sun et al. found that mutations in the S gene of PEDV wild strain PEDV-LY4-98 resulted in increased pathogenicity to neonatal piglets [29]. Additionally, the S protein is highly immunogenic, which makes it a useful target for the development of effective vaccines against coronavirus [30]. 

Although there are studies describing PDCoV isolated in China, much more information is needed about their emergence and circulation if we are to better understand the evolution and epidemiology of PDCoV in China. For example, China ranks first in pork production in the world and Sichuan province in southwest China ranks first in pork production in China, and yet, to date no information on PDCoV emerging from or circulating in this area is accessible except for a submitted sequence (CH/Sichuan/S27/2012). In this study, we describe PDCoV strain CHN-SC2015, the first report of a strain isolated from this area. Sequence alignment, phylogenetic and recombination analysis based on the complete genome were performed to determine genetic variabilities and evolutionary relationships within this species. The pathogenicity of this strain to five-day-old piglets was investigated by clinical assessment and histology. 

## 2. Material and Methods

### 2.1. Clinical Samples

From two farms with intensive pig operations in Sichuan province, the intestinal contents from ten suckling piglets with diarrhea were collected from 2015 to 2017. The contents from all the piglets proved to be PDCoV-positive by RT-PCR of the M gene. Prior to virus isolation, the samples were homogenized in 5 mL of Dulbecco’s modified Eagle’s medium (DMEM) (Gibco, Carlsbad, CA, USA) supplemented with 1% antibiotic-antimycotic (Solarbio, China), subjected to three cycles of freezing and thawing, then centrifuged at 5000 rpm for 20 min at 4 °C. Supernatants were filtered through a 0.45 µm sterile filter and stored at −80 °C. 

### 2.2. Cells and Antibodies

LLC porcine kidney cells (LLC-PK) were maintained in DMEM supplemented with 10% fetal bovine serum (FBS) (PAN, Germany) and 1% antibiotic-antimycotic at 37 °C in a humidified atmosphere of 5% CO_2_. The rabbit anti-PDCoV N monoclonal antibody was prepared in our laboratory. FITC-conjugated goat anti-rabbit IgG was purchased from Bioss. 

### 2.3. Virus Isolation and Propagation

For virus isolation, LLC-PK cells at 90% confluence in T25 flasks were washed three times with DMEM supplemented with 1% antibiotic-antimycotic and 7.5 µg/mL trypsin-EDTA (henceforth referred to as maintenance media) then inoculated with 1 mL of the filtered inoculums. The virus was allowed to adsorb 2 h at 37 °C, then 7 mL of maintenance medium was added to each flask. The cells were cultured continuously at 37 °C until a cytopathic effect (CPE) was observed. The infected cell suspensions were subjected to three cycles of freezing and thawing then centrifuged at 8000 rpm for 10 min at 4 °C. Supernatants were collected and stored at −80 °C for the subsequent passage. 

For subsequent passage, LLC-PK cells at 90% confluence in T25 flasks were washed three times with maintenance media and then inoculated with 1 mL of CHN-SC2015. After incubation for 2 h at 37 °C, 7 mL of maintenance medium was added to each flask. Upon a >80% CPE being observed, the flasks were subjected to three cycles of and thawing then centrifuged at 8000 rpm for 10 min at 4 °C. Supernatants were collected and stored at −80 °C.

### 2.4. TCID_50_ Assay

TCID_50_ assay was performed using LLC-PK cells according to the method described by Dong et al. [13]. Briefly, the confluent cell monolayers seeded in 96-well plates were washed twice with maintenance medium and then inoculated with 100 µL of 10-fold serially diluted PDCoV; At each dilution, there were eight technical replicates. An amount of 150 µL of maintenance medium was added to each well after the cells and the virus had incubated for 1.5 h at 37 °C. The CPE was observed for 4 days and was analyzed by the methods of Reed & Muench [31].

### 2.5. Immunofluorescence Assay (IFA)

LLC-PK cells in 12-well plates were infected with PDCoV at a multiplicity of infection (MOI) of 0.04. After 24 h, the cells were rinsed then fixed with 4% paraformaldehyde for 30 min at room temperature. Cells were rinsed again and permeabilized with 0.5% Triton-X-100 for 30 min at room temperature, then washed three times with phosphate-buffered saline (PBS) for 5 min and blocked with 2% bovine serum albumin (BSA) for 1 h at 37 °C. Cells were then incubated for 16 h at 4 °C with rabbit anti-PDCoV N monoclonal antibody (1:200), washed again and incubated with FITC-conjugated goat anti-rabbit IgG (1:200) for 1 h at room temperature. Finally, cells were treated with 4′,6-diamidino-2-phenylindole (DAPI) for 10 min, rinsed, mounted on glass slides and observed with the fluorescence microscope (IX73, Olympus, Japan).

### 2.6. Electron Microscopy

Cultures of PDCoV-infected LLC-PK cells were harvested when CPE was observed, subjected to three cycles of freezing and thawing, then centrifuged at 5000 rpm for 20 min at 4 °C. Supernatants were filtered through 0.45 µm sterile filters then centrifuged at 50,000 rpm for 4 h at 4 °C using the ultracentrifuge MTX150 (Thermo Fisher Scientific, Waltham, MA, USA). Viral particles were resuspended in PBS, negatively stained with 2% sodium phosphotungstic acid and examined with a transmission electron microscope (JEM-1200EX, Olympus, Japan).

### 2.7. RT-PCR/qRT-PCR

Total RNA was extracted from PDCoV-infected LLC-PK cell cultures using a UNlQ-10 Column TRIzol Total RNA Isolation Kit (Sangon, China), then was subjected to RT-PCR/qRT-PCR using primers specific to the S and M gene of the PDCoV. The primer sequences used for RT-PCR to amplify S gene are 5′-CACCAGGACGCCTTCTTGTGAGG-3′ and 5′-CTACCATTCCTTAAACTTAAAGGACG-3′. PrimeSTAR Max Premix (Takara, China) was used for RT-PCR reactions and the amplification conditions were 98 °C for 5 min, then 32 cycles of 98 °C for 30 s, 56 °C for 30 s, and 72 °C for 45 s. The PCR products were sequenced by the Sangon company and aligned using DNAMAN 7.0 software. Primer sequences used for qRT-PCR are 5′-CCAATGGGTACATGGAGGT-3′ and 5′-GTGGCGGATTTCTAACTGA-3′ SYBR Green I Master Mix (Takara, China) was used for the qRT-PCR reactions and the amplification conditions were 95 °C for 30 s, then 40 cycles of 95 °C for 5 s, 55 °C for 30 s, and 72 °C for 30 s using the LightCycler 96 system (Roche, Germany). A standard curve was constructed using 10-fold serial dilutions of pMD18-T containing the PDCoV M gene sequence; the quantity of PDCoV RNA was calculated based on the standard curve and expressed as the copies per milliliter of cell culture. The primer sets were designed using the CH/Sichuan/S27/2012 sequence as a template (GenBank accession No. KT266822.1) and were synthesized by the Sangon company. 

### 2.8. Viral Replication Kinetics

Confluent LLC-PK cells in T75 flasks were infected with PDCoV (MOI = 0.04). After adsorption for 1.5 h at 37 °C, the inoculum was removed and 25 mL of the maintenance medium was added to each flask. Culture supernatants were collected at 6, 12, 24, 36, 48, and 60 h post-infection and the quantity of viral RNA was determined using qRT-PCR.

### 2.9. Genetic Evolution and Mutation Analysis

The complete genome sequence of the PDCoV strain CHN-SC2015 from the passage 12 (10^6.35^ TCID_50_/mL) was determined by next-generation sequencing (NGS) technology using an Illumina 1.9 platform. Sequences were mapped to the known porcine deltacoronavirus strain CH/Sichuan/S27/2012 and were assessed using the quality control tool FastQC (http://www.bioinformatics.babraham.ac.uk/projects/fastqc/). Sequence alignment, based on the complete genome of the PDCoV strain CHN-SC2015 and 59 available strains deposited in GenBank, was performed using ClustalW in MEGA X software. Phylogenetic analysis of CHN-SC2015 and the 59 reference strains were analyzed using MegAlign in DNAStar Lasergene Version 7. Phylogenetic trees were constructed based on the sequences of the complete genome and the S gene using the maximum likelihood method in MEGA X with a bootstrap analysis of 1000 replicates and visualized with iTOL (https://itol.embl.de/). The aligned sequences were also analyzed using Recombination Detection Program 4 (RDP4) [32] and Simplot 3.5.1 [33] to predict the possible recombination events in PDCoV strain CHN-SC2015. 

### 2.10. Pathogenicity of PDCoV Strain CHN-SC2015 in Piglets

Seven five-day-old piglets were randomly divided into two groups; five piglets in the experimental group and two in the negative control group. Fecal samples from all the piglets tested negative for PEDV, TGEV, classical swine fever virus (CSFV), porcine reproductive and respiratory syndrome virus (PRRSV), porcine pseudorabies (PRV), porcine group A rotavirus (PoRV), and porcine circivirus 2 (PCV2) prior to virus infection. The experimental piglets were inoculated orally with 10 mL of DMEM containing 10^6.35^ TCID_50_/mL PDCoV CHN-SC2015 from passage 12, the control piglets received 10 mL of maintenance medium orally; experimental and control piglets were housed in separate rooms. All the piglets were monitored daily for clinical signs of infection, including diarrhea, vomiting, and lethargy and all observations were recorded. Three challenged piglets and one control piglet were necropsied at 5 days post-infection (dpi), the remaining piglets were necropsied at 8 dpi. After necropsy, jejuna were fixed in 4% formaldehyde and prepared for histological examination using hematoxylin and eosin (HE) staining. 

### 2.11. Statistical Analysis

The experiments were performed in triplicate. Data are shown as the mean ± standard deviation (SD). A one-way ANOVA test was used to measure significant differences between groups. *p* values < 0.05 were considered statistically significant.

### 2.12. Ethics Statement

The authors confirm that the ethical policies of the journal, as noted on the journal’s author guidelines page, have been adhered to and the appropriate ethical review committee approval has been received. The animal experiment was approved by the Institutional Animal Care and Use Committee of Sichuan Agricultural University (IACUC#RW2016-090, approval date: 8 September 2016). 

## 3. Results

### 3.1. Virus Isolation and Identification

**Figure 1:**
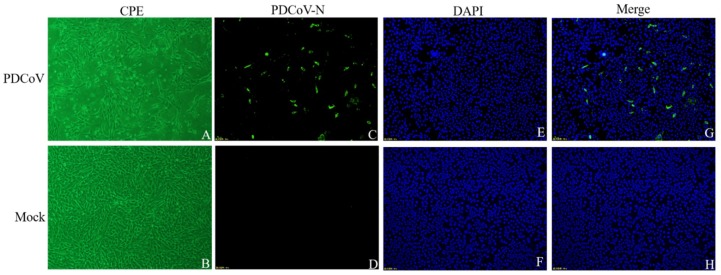
Isolation and identification of PDCoV CHN-SC2015 in LLC-PK cells by cytopathic effect (CPE) and immunofluorescence assay (IFA). The confluent LLC-PK cells were infected with PDCoV CHN-SC2015 and the CPE was recorded at 24 h post-infection. (**A**): Micro examination of the PDCoV-infected LLC-PK cells; (**B**): Micro examination of the mock-infected LLC-PK cells. (**C**–**H**): LLC-PK Cells were fixed and stained with rabbit anti-PDCoV N protein and FITC-goat anti-rabbit IgG at 24 h post-infection (green); nuclei were counterstained with DAPI (blue). Magnification = 10×.

PDCoV was successfully isolated from one sample collected from sick piglet, namely PDCoV CHN-SC2015. The CPE of PDCoV CHN-SC2015 in LLC-PK was characterized by cell clustering and rounding, then death 24 h after infection (Figure 1A). Mock-infected cells (Figure 1B) remained flat and attached to the dish. IFA, at 24 h post-infection, was used to visualize the presence of the N protein in PDCoV CHN-SC2015. As shown in Figure 1C–H, green fluorescence, from FITC-anti-N antibodies, was observed in infected cells; none was observable in mock-infected cells. 

**Figure 2:**
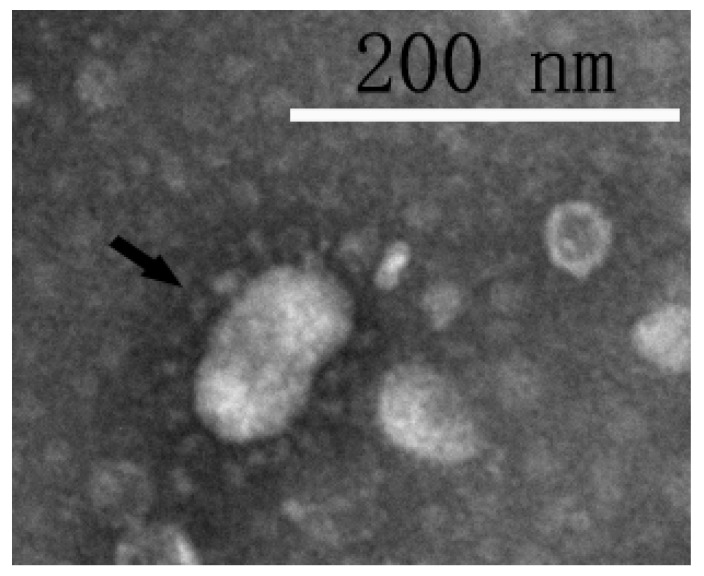
Electron microscopy of PDCoV strain CHN-SC2015. The virus particles were resuspended in PBS and negatively stained with 2% sodium phosphotungstic and examined with a transmission electron microscope. The arrow indicates the crown-shaped projections on the virus surface. Bar: 200 nm.

Viral particles negatively stained with 2% sodium phosphotungstic acid were examined by EM. Visible in Figure 2 are the crown-shaped projections on the surface periphery, which is the typical topology of coronavirus. The particle diameter is about 60–120 nm. These results demonstrate that the PDCoV CHN-SC2015 was successfully isolated from the intestinal contents of the diarrheal piglet. 

### 3.2. Viral Replication

To further characterize the growth of strain CHN-SC2015 in LLC-PK cells, TCID_50_ and qRT-PCR assays were performed every fifth passage for 30 passages (Table 1). Viral titers increased approximately 1000-fold between the fifth and 10th passages, then increased 10-fold and held relatively steady through the 30th passage. Viral RNA/ µL increased about 10-fold by the 25th passage then fell off slightly. These data demonstrate that PDCoV strain CHN-SC2015 actively propagates in LLC-PK cells and that this cell line is suitable for PDCoV isolation and propagation.

### 3.3. Viral Replication Kinetics in LLC-PK Cells

**Figure 3:**
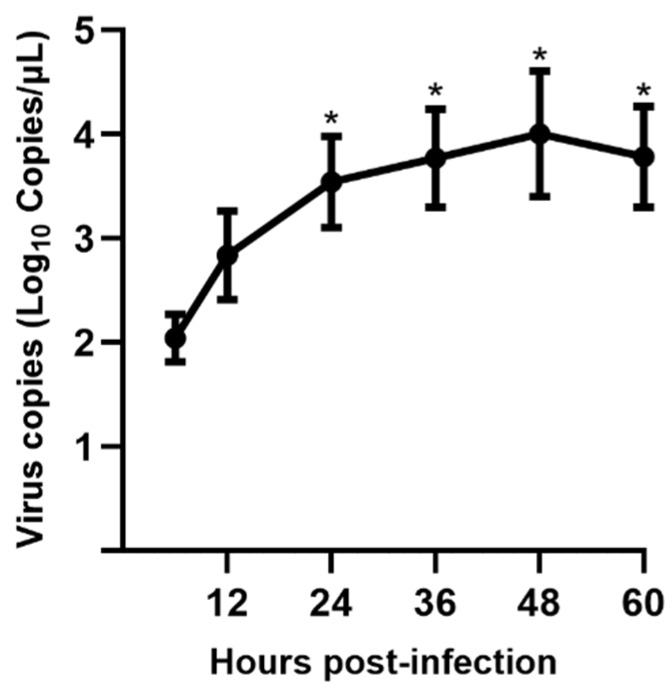
PDCoV CHN-SC2015 replication kinetics in LLC-PK cells was determined by qRT-PCR. The total viral RNA from PDCoV CHN-SC2015 infected LLC-PK cells at different times (12 h, 24 h, 36 h, 48 h and 60 h) was extracted and subjected to qRT-PCR. The quantity of PDCoV viral RNA was calculated based on the standard curve and is expressed as the copies per milliliter of the cell culture. * indicates that the significant difference compared to 6 h post-infection.

qRT-PCR was used to characterize the replication kinetics of CHN-SC2015 passage 17 in LLC-PK cells. RNA was extracted from PDCoV CHN-SC2015 infected LLC-PK cells at different times during a 60-h infection time course. As shown in Figure 3, viral RNA copies gradually increased until peaking at 48 h post-infection, then fell off slightly at 60 h post-infection.

### 3.4. Sequence Variation in the S Gene During Serial Passage

To identify and analyze variations in the S gene of strain CH-SC2015 during serial passage, we amplified the S gene from the original isolate and at every fifth passage by RT-PCR. The nucleotide and amino acid sequences at every passage were compared against the parental sequences using DNAMAN 7.0 (Table 2). Nucleotide changes were observed at positions 484, 500, 578, 787, 946, 1188, 1443, 1450, 1587, 1922, and 2636. All of these resulted in amino acid substitutions, except for the synonymous mutations at positions 1443 and 1587 (indicated in boldface). Four nucleotide mutations had occurred by passage 5, by passage 30, ten nucleotides were different than parental. Amino acid 162 (indicated in boldface) is a glycosylation site on the surface of the S protein.

### 3.5. Genomic Characterization of PDCoV Strain CHN-SC2015

**Figure 4:**
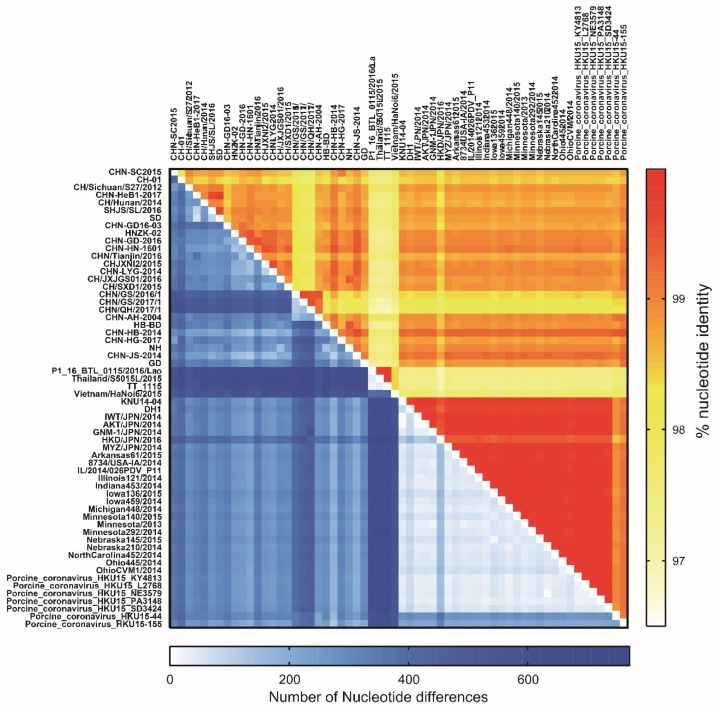
Heat map of nucleotide identities and differences between CHN-SC2015 and 59 reference strains in GenBank. The level of nucleotide identity (top right) and the number of nucleotide differences (bottom left) between CHN-SC2015 and other 59 reference strains were analyzed using Clustal W software in MEGA X. The nucleotide identity and difference are represented by a gradation of color; the deeper the color, the greater the identity or difference.

**Figure 5:**
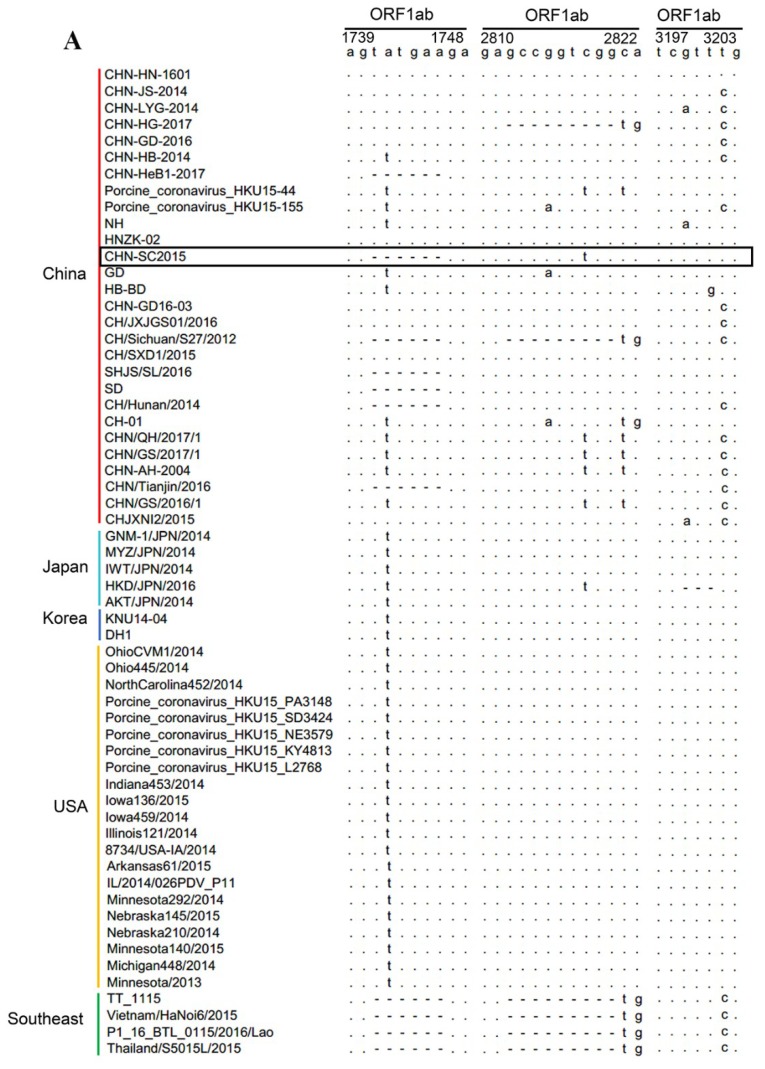

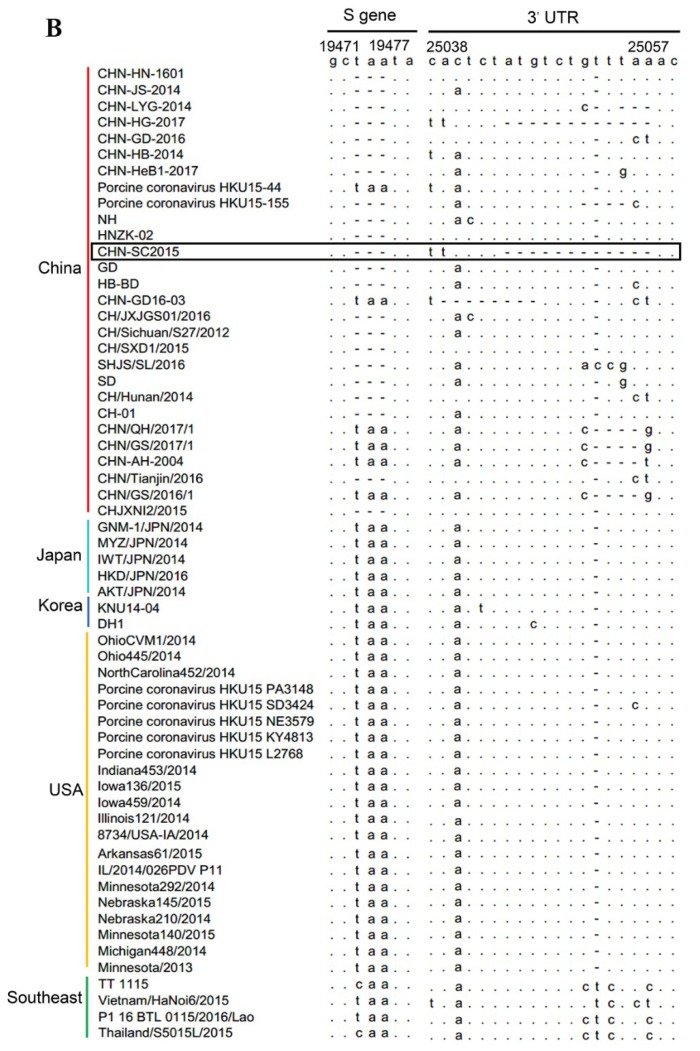
Analysis of deletions and insertions in the complete genome of PDCoV strain CHN-SC2015. Alignments, ORF1ab gene (**A**) and the S gene and 3′ UTR (**B**) were performed with ClustalW software in MEGA X. PDCoV strain CHN-SC2015 is outlined with a box. A dash indicates that the deleted nucleotide is present in the other 59 sequences compared to the consensus sequences.

The complete genome sequence of PDCoV strain CHN-SC2015 is 25,403 nt and was deposited at DDBJ/EMBL/GenBank under accession number MK355396. We compared CHN-SC2015 with 59 other PDCoV strains available in GenBank and found that the level of nucleotide identities ranged from 97.5% to 99.1%; the number of differences in the nucleotide sequences ranged from 606 to 230 (Figure 4, Table 3). The sequence alignment of the CHN-SC2015 strain revealed a 3-nt deletion (TAA) in the S gene compared to the US, Japan, South Korea, and Southeastern Asia PDCoV strains. This deletion was also present in a number of other Chinese PDCoV strains, indicating that they may have evolved from a common ancestor (Figure 5B). CHN-SC2015 also contained a 6-nt deletion (TATGAA) and a 9-nt insertion (GCCGGTCGG) in the ORF1ab gene compared with the PDCoV strains from Lao PDR, Thailand, and Vietnam, and a 3-nt deletion (GTT) compared with HKD/JPN/2016 (Figure 5A). Additionally, a 11-nt deletion was found in the 3′UTR, not seen in 58 of the other PDCoV strain; PDCoV strain CHN-HG2017 from China also contained this 11-nt deletion (Figure 5B).

### 3.6. Phylogenetic and Recombination Analysis of PDCoV Strain CHN-SC2015

**Figure 6:**
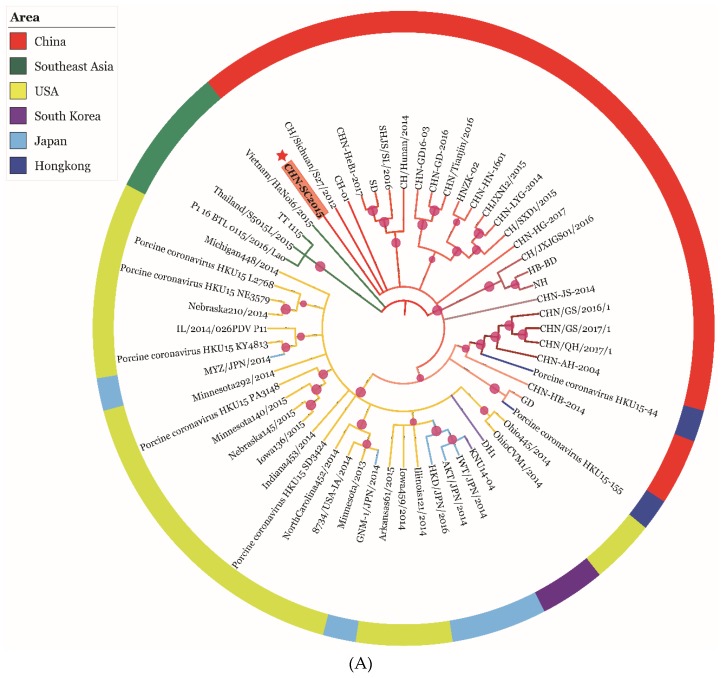

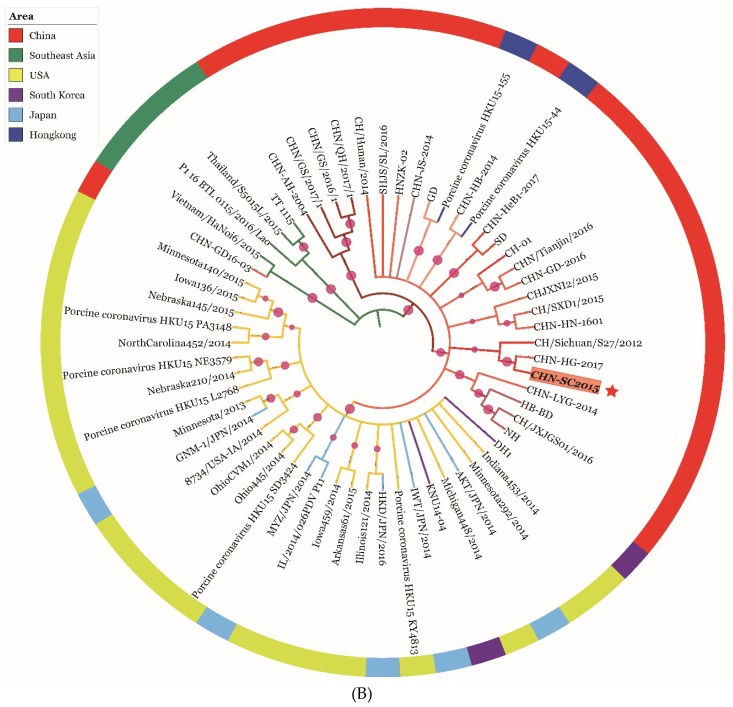
Phylogenetic trees based on the complete genome (**A**) and S gene (**B**) of PDCoV strain CHN-SC2015. Phylogenetic trees were constructed using the neighbor-joining method in MEGA7. CHN-SC2015 is indicated with a red asterisk. The branches in red, green, yellow, purple, light blue, and dark blue represent the clades from China, Southeast Asia, USA, South Korea, and Hong Kong, respectively. The trees were constructed using the ML method and a bootstrap analysis (*n* = 1000) in Mega X software. Bootstrap values > 50% are shown at the branch points.

The phylogenetic analysis was based on sequences of the complete genome and the S gene of CHN-SC2015 and the 59 reference strains. An analysis of the complete genome (Figure 6A) indicated that the CHN-SC2015 isolates were more closely related to the other PDCoV strains in China than to the strains from Southeast Asia, USA, Japan, and South Korea, indicating the diversity of genetic relationships and regional and epidemic characteristics among these strains. CHN-SC2015 did not cluster with Southeast Asia strains. When compared with the Chinese strains, CHN-SC2015 was grouped into a separate novel subcluster. An analysis of S genes revealed that PDCoV CHN-SC2015 clustered with CHN-HG-2017; there is a 3-bp deletion in CHN-HG-2017 and also present in CHN-SC2015 (Figure 5 and Figure 6B). 

**Figure 7:**
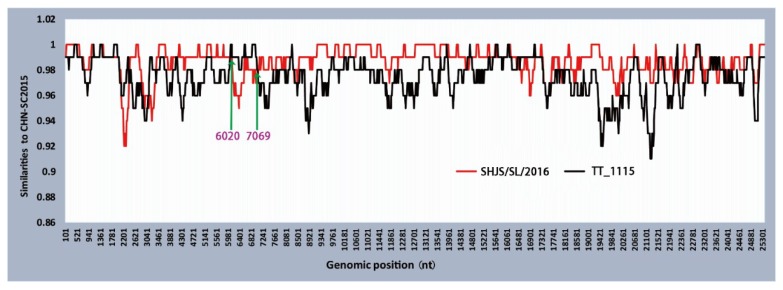
Recombination analysis of the complete genome of PDCoV strain CHN-SC2015. Potential recombination events were identified using Recombination Detection Program 4 (RDP4) and then examined using similarity plots and bootstrap analysis in Simplot 3.5.1. in the case of *p*-values less than 10^−8^. Major and minor parents are represented by red and black, respectively. The green arrows indicate the recombination breakpoints.

To evaluate the recombination events of PDCoV CHN-SC2015 during the process of evolution, its complete genome sequence was analyzed using RDP4 and Simplot 3.5.1. As shown Figure 7, CHN-SC2015 potentially underwent recombination with major parent SHJS/SL/2016 (GenBank accession number: MF041982) and minor parent TT_1115 (GenBank accession number: KU984334) (*p* < 0.01). The predicted recombination breakpoints in CHN-SC2015 are located between nucleotides 6020 and 7069, the region encoding nsp3 and nsp4. 

### 3.7. Pathogenicity of PDCoV Strain CHN-SC2015 in Piglets

**Figure 8:**
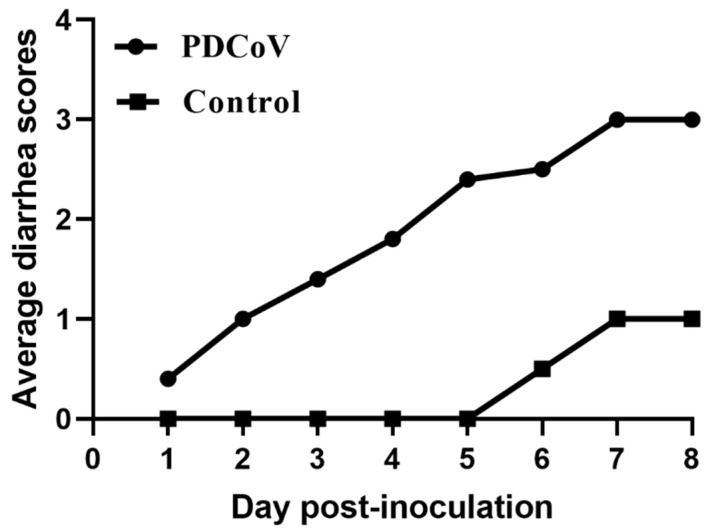
Diarrhea scores of the challenged piglets. The five-old-day piglets were inoculated orally with PDCoV CHN-SC2015 passage 12 and DMEM. Clinical signs were observed daily, and diarrhea was scored for challenged piglets as followed: 0 = normal feces,1 = soft, 2 = liquid with some solid content,3 = watery with no solid content.

**Figure 9:**
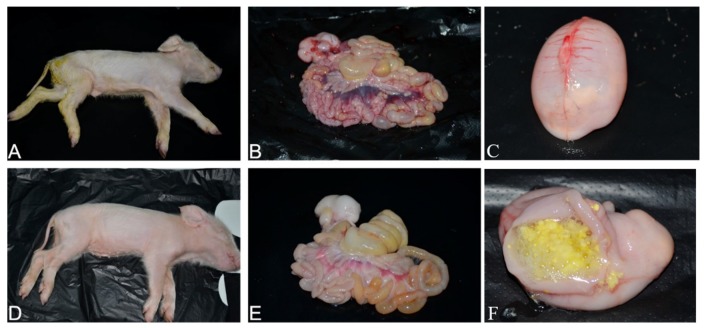
Pathology of PDCoV strain CHN-SC2015 in five-day-old piglets. The images are representative of experimental and control piglets. Clinical observation of a piglet challenged with PDCoV CHN-SC2015 passage 12 (**A**) or DMEM (**D**) at 5 dpi. Macroscopic lesions of gastrointestinal tissue from the infected piglet (**B**) and uninfected (**E**) at 5 dpi. Macroscopic lesions of the stomach from the infected piglet (**C**,**F**) at 5 dpi.

The pathogenicity of cell-cultured PDCoV strain CHN-SC2015 from passage 12 was demonstrated in the piglets. The daily clinical observations are shown in Table 4; one challenged piglet displayed mild diarrhea at 1 dpi and the other piglets developed mild or watery diarrhea at 3–5 dpi (Table 4, Figure 8 and Figure 9A). Lethargy and anorexia were developed in all the piglets by 2 dpi.

**Figure 10:**
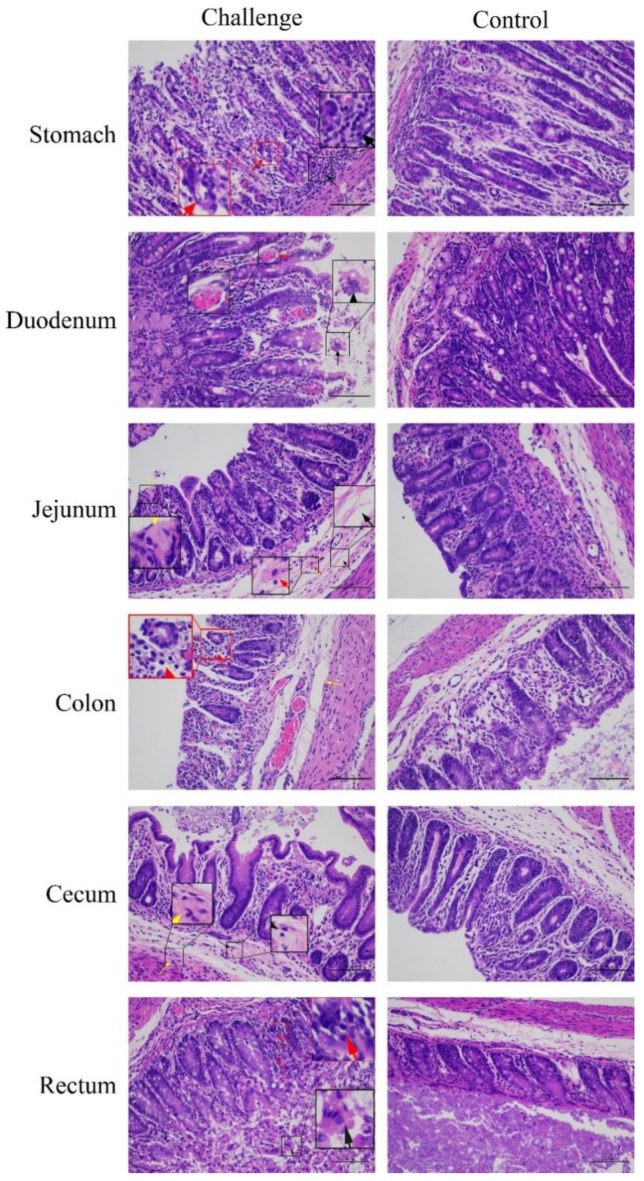
Histological analysis of gastrointestinal sections and stomach from piglets infected with PDCoV CHN-SC2015. The tissues from the piglets incubated with CHN-SC2015 or DMEM were collected at 5 DPI and were stained with hematoxylin and eosin. The different colors of arrows and boxes indicated the typical histological lesion in the different tissues. Scale bars are also shown in each picture.

**Figure 11:**
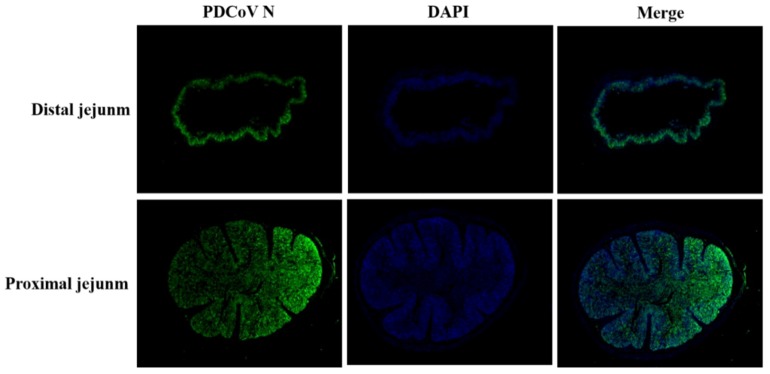
PDCoV N-protein was detected in sections of jejunum by immunofluorescence staining. Jejunum samples from five-old-day piglets infected with PDCoV CHN-SC2015 were fixed and then stained with the rabbit anti-PDCoV N protein monoclonal antibody and FITC-conjugated goat anti-rabbit IgG (green); the nucleus was counterstained with DAPI (blue). Magnification = 2×.

The gastrointestinal and non-gastrointestinal tissues of infected piglets were collected and analyzed. By gross observation, the infected piglets exhibited thin and transparent intestinal walls and had accumulated large amounts of yellow fluid in the intestinal lumen at 5 dpi (Figure 9B). The stomachs contained coagulated milk (Figure 9C,F) and mesenteric congestion was also observed (Figure 9B). No obvious lesions were observed in the control piglets (Figure 9D,E). Upon histopathological analysis of the gastric glandular and intestinal mucosa, gastric epithelial cells and villous enterocytes were necrotic and sloughing into the lumen. Numerous inflammatory cells infiltrates were absent in the intestinal cavity and lamina propria, which occasionally contained congested blood vessels (Figure 10). The PDCoV N protein was detected in the distal and proximal sections of the jejuna of infected piglets as can be seen in Figure 11. These results demonstrate that PDCoV strain CHN-SC2015 is highly pathogenic in piglets.

### 3.8. Viral Shedding and Distribution

**Figure 12:**
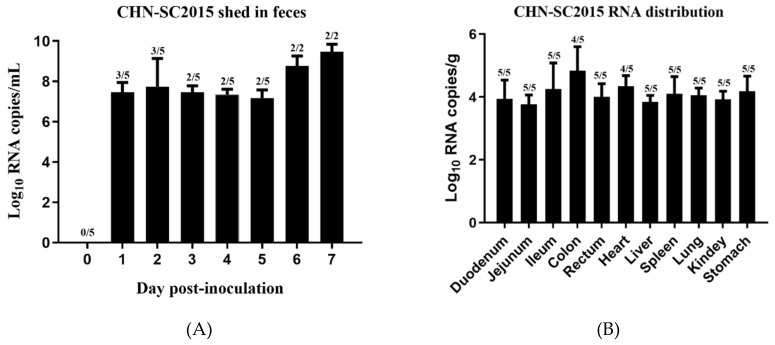
Viral shedding and distribution in the challenged piglets. (**A**) The five-day-old piglets were infected with PDCoV CHN-SC2015 and viral shed in feces was quantitated daily from two or three of the challenged piglets. (**B**) The five-day-old piglets were infected with PDCoV CHN-SC2015 and distribution of CHN-SC2015 in the intestines and internal organs was assayed after necropsy.

To determine viral shedding during the days post-infection, qRT-PCR was used to quantify viral RNA from rectal swabs. Viral distribution in the intestines and internal organs was also determined using qRT-PCR. As shown Figure 12A, virus was shed in feces from 1 dpi to 5 dpi at about the same level, at 6 and 7 dpi the number of viral RNA copies rose about 100-fold to10^9.43^ copies/mL of rectal swab sample. Viral RNA was detected in duodenums (average 10^3.94^ copies/g), jejunums (average 10^3.77^ copies/g), ileums (average 10^4.25^ copies/g), colons (average 10^4.83^ copies/g), and rectum (average 10^4.01^ copies/g). It was also detected in hearts (average 10^4.38^ copies/g), livers (average 10^3.89^ copies/g), spleens (average 10^4.14^ copies/g), lungs (average 10^4.04^ copies/g), kidneys (average 10^3.81^ copies/g), and stomachs (average 10^4.27^ copies/g) (Figure 12B), indicating that CHN-SC2015 has broad tissue tropism in five-day-old piglets. No PDCoV RNA was detected in the control swine. 

## 4. Discussion

Coronaviruses are enveloped RNA virus in the *Coronaviridae* family [34] and to date, they have been classified into four genera (α, β, γ and δ). Several alphacoronaviruses, such as PEDV and TGEV cause diarrhea and vomiting in piglets, leading to enormous economic losses in the swine industry [35,36]. Swine enteric coronavirus (SeCoV), a recombinant between PEDV and TGEV reported in 2016, also causes diarrhea in swine [37]. Additionally, some novel alphacoronaviruses related to the bat coronavirus HKU2 have recently been reported in China; these viruses, porcine enteric alphacoronavirus (PEAV) [38], swine enteric alphacoronavirus (SeACoV), [39] and swine acute diarrhea syndrome coronavirus (SADS-CoV) [40] are also major pathogens of diarrheal disease in swine. The deltacoronavirus, PDCoV, also causes diarrhea, vomiting, and dehydration in nursing piglets, and it is clinically indistinguishable from other enteric diseases caused by these porcine enteropathogenic coronavirus. Moreover, coexistence of pathogens, such as PDCoV and PEDV, PDCoV and PoRV, increase the mortality rate of infected piglets, which incurs massive economic losses to porcine industries [41]. Taken together, the pathogens associated with diarrhea in piglets have attracted much needed attention to the necessity for expanded preventative, diagnostic, and treatment measures.

In this study, PDCoV was purified from fecal samples from 10 diarrheal, PDCoV-positive piglets then passed in LLC-PK cells. From the 10 samples, only the PDCoV strain CHN-SC2015 was successfully isolated. Viral identification was done by RT-PCR/qRT-PCR, IFA, and EM. Sample quality is a crucial factor for the successful isolation of PDCoV. Clinical samples may contain unknown viruses or other pathogens or very low amounts of infectious virus, all making successful isolation difficult [18]. The addition of exogenous trypsin in vitro enhances virus entry into the host cells by proteolytic cleavage of the S protein, which is necessary for coronavirus infection [42]. Therefore, the concentration of trypsin in the LLC-PK cell culture media may contribute to the isolation of PDCoV [43]. In our study, the maintenance medium was supplemented with 7.5 µg/mL trypsin, which is higher than the concentrations used in the studies that demonstrated the effectiveness of trypsin in in vitro PDCoV infections [44]. In addition, the time at which the sample is actually collected may also contribute to the success of PDCoV isolation.

Currently, several cell lines have been found to be suitable for PDCoV infection. Zhu et al. [45] and Jung et al. [43] reported that the porcine intestinal epithelial cells, IPI-2I, and IPEC-J2 cells, are susceptible to PDCoV infection. Li et al. [46] found that the PDCoV infect cells of galline and human origin, such as galline hepatoma (Leghorn male hepatoma, LMH), DF-1 fibroblast, human hepatoma (Huh7), and Hela cells [45,46]. Several PDCoV strains have also been successfully propagated in LLC-PK cells and swine testicular (ST) cells [20]. In our study, LLC-PK cells were used to propagate PDCoV CHN-SC2015 to a high titer (10^8.22^ TCID_50_/mL at passage 20). 

Mutations in the coronavirus S protein are considered the key factors responsible for PDCoV virus pathogenesis and host tropism change [47,48]. A sequence analysis based on the complete genome of the PDCoV strain CHN-SC2015 showed a 3-nt deletion in the S gene; this deletion is found in other Chinese isolates but not in isolates from the USA, Japan, South Korea, or Southeast Asia, suggesting a mechanism for differences in virulence among these PDCoV strains. However, whether this particular deletion contributes to the efficiency of viral replication and/or the virulence of these strains is in question. After passage 30, we found six nucleotide changes in the S gene compared with the original sample CHN-SC2015, which induced corresponding amino acid changes, excluding the synonymous mutations at the position 1443 and 1587. The amino acid mutation at position 162 is a glycosylation site located on the surface of amino-terminal domain within the S protein. The lack of glycosylation at this site may hinder the virus’ ability to evade the host’s immune response because of the lack of glycan shielding of S protein epitopes [34,49]. The 11-bp deletion in 3′UTR in this strain was not found in 58 of the reference strains; the exception was PDCoV strain CHN-HG-2017, indicating that CHN-SC2015 may be a novel strain to the province of Sichuan. According to the phylogenetic results, based on the complete genome and S gene, PDCoV CHN-SC2015 falls into a different clade compared with many other PDCoV strains currently circulating in China. This difference indicates that variations, for the most part, existed in other genes of the PDCoV during evolution and transmission [50]. Moreover, CHN-SC2015 belongs to a different clade than PDCoV isolates from Lao PDR, Thailand, and Vietnam. 

Recombination events have been reported in animal and human coronaviruses, such as PEDV and severe acute respiratory syndrome (SARS), these viruses have a history of recombination with other lineages of coronavirus [51,52]. Su S et al. posit that the large genome and multiple host species were major factors for the high rate of recombination events, which can alter tissue tropism, transmission routes, and host specificity [53]. Like most coronaviruses, deltacoronavirus also possess the potential to undergo some recombination events that can facilitate interspecies transmission and jump to the new hosts. Lau et al. reported that porcine coronavirus HKU15 from recombination between sparrow coronavirus HKU17 and bulbul coronavirus HKU11 in the S gene region [17]. In this study, a recombination analysis predicted that CHN-SC2015 had undergone recombination with strain SHJS/SL/2016 from the China and TT_1115 from Vietnam. The predicted breakpoint is located in the *nsp3* gene, which is similar to the strain CHN-HG-2017, which may have been derived from the major parent strain SXD1 from China and minor parent strain HaNoi6 from Vietnam [54]. However, the breakpoint of the recombinant virus between the two different strains may be distinct.

In recent years, the pathogenicity of PDCoV to piglets has become a focus of investigation [16,55,56]. The clinical profile of PDCoV infection, diarrhea, vomiting, dehydration, and death, are indistinguishable from other enteropathogenic porcine coronavirus infections such as PEDV and TGEV [19]. In our study, the pathogenicity of PDCoV strain CHN-SC2015 was demonstrated in five-day-old pigs orally inoculated with 10^6.35^ TCID_50_/mL cell-culture-adapted virus. Gastrointestinal tissues from a challenged piglet at 5 dpi showed evident lesions indicating that the intestinal tract is the main site for virus replication. Recent reports have found that PDCoV is transmissible by indirect contact with an infected piglet, indicating that the respiratory tract may be the minor target for PDCoV infection [57]; Moreover, in our study, we also found that the control piglets had developed mild diarrhea along with PDCoV CHN-SC2015 infection, leading to a low score of diarrhea from 6 DPI, which further supports the notion that PDCoV can transmit via the respiratory tract. This route of infection is also true for PEDV, which can disseminate from the nasal cavity to the intestinal mucosa, providing further evidence for the airborne transmission of gastrointestinal coronaviruses [58]. Analysis of daily fecal samples and post-mortem tissues and organs by qRT-PCR demonstrated that PDCoV RNA continually and gradually increased from 1 to 7 dpi in feces, PDCoV RNA was distributed in multiple tissues and organs, indicating extensive tissue tropism; these results are consistent with other studies [18,59].

In summary, CHN-SC2015 is the first PDCoV strain isolated from southwest China, the country’s largest pig-producing area. The phylogenetic and recombination analyses indicate that CHN-SC2015 is closely related to the other PDCoV strains in China than to the strains from Southeast Asia, USA, Japan, and South Korea. CHN-SC2015 contained deletions and/or insertions in ORF1ab, S, and the 3′UTR compared with the strains available in GenBank. Infection of five-day-old piglets with PDCoV CHN-SC2015 demonstrated its pathogenicity. Our results enrich the body of information on the molecular epidemiology and pathogenic of PDCoV. 

## Figures and Tables

**Table 1:** Replication of early and late passages of PDCoV strain CHN-SC2015.

Isolate	Parameter		Results of Different Passage					
		P1	P5	P10	P15	P20	P25	P30
CHN-SC2015	CPEs	+	+	+	+	+	+	+
	qRT-PCR C_T_	ND	22.09	24.01	18.71	18.44	18.15	20.02
	Virus RNA Copies (Log_10_ Copies/μL)	ND	1.75	1.19	2.74	2.82	2.90	2.36
	Infectious titer (Log_10_ TCID_50_/mL)	5.26	4.31	7.00	7.53	8.22	7.53	8.11

Note: + indicated that the CPEs were observed in LLC-PK cells. ND, not done.

**Table 2:** Sequence variation analysis of the S gene during serial passage.

Passage	Nucleotide Position											Amino Acid Position								
	484	500	578	787	946	1188	1443	1450	1587	1922	2636	162	167	193	263	316	396	484	641	879
parent	T	A	A	G	A	T	**A**	G	**C**	A	T	**Y**	H	D	V	N	N	E	Q	L
P5	G	A	A	G	G	T	**A**	G	**T**	G	T	**D**	H	D	V	D	N	E	R	L
P10	T	C	A	G	G	T	**A**	G	**T**	G	C	**Y**	P	D	V	D	N	E	R	S
P15	G	A	A	G	G	T	**A**	C	**T**	G	C	**D**	H	D	V	D	N	Q	R	S
P20	T	C	A	G	G	T	**A**	C	**T**	G	C	**Y**	P	D	V	D	N	Q	R	S
P25	T	C	A	T	G	T	**A**	C	**T**	G	C	**Y**	P	D	L	D	N	Q	R	S
P30	T	C	C	T	G	A	**G**	C	**T**	G	C	**Y**	P	A	L	D	K	Q	R	S

Note: Parent = original sample. A/G at position 1443 and C/T at position 1587 are the synonymous mutation sites. Y/D at position 162 is the glycosylation site.

**Table 3:** Nucleotide and amino acid identity of CHN-SC2015 compared to 59 reference strains in GenBank.

Area	Nucleotide and Amino Acid Identity (%)								
	Complete Genome	ORF1a	ORF1ab	S	E	M	NS6	N	NS7
China	97.7–99.1	98.0–99.1/ 98.0–99.4	98.1–99.1/ 98.0–99.6	97.0–99.3/ 96.0–99.5	96.4–100/ 95.2–100	97.7–99.8/ 96.8–99.5	96.5–100/ 92.6–100	97.1–99.9/ 95.3–100	97.5–100/ 94.0–100
Japan	97.9–98.4	98.0–98.5/ 92.6–99.2	98.3–98.7/ 95.5–99.4	97.6–98.3/ 97.8–98.6	99.2–99.6/ 100	98.5–98.8/ 99.1	98.2–98.9/ 96.8–97.9	98.7–99.2/ 99.7–100	99.2–99.5/ 97.5–98.5
South Korea	98.5–98.6	98.4–98.5/ 99.1–99.2	98.6/ 99.3–99.4	98.1–98.3/ 98.2–98.6	99.6/100	98.6/99.1	98.9/ 97.9–98.9	99.0/100	99.2–99.3/ 97.5–98
USA	98.1–98.6	98.4–98.5/ 99.0–99.2	98.6–98.7/ 99.3–99.4	98.1–98.3/ 98.1–98.6	99.2–100/ 100	98.3–98.8/ 98.6–99.1	98.6–99.3/ 97.9–98.9	98.9–99.2/ 99.4–100	99.2–99.5/ 97.5–99.0
Southeast	97.5–98	97.8–98.0/ 98.2–98.6	97.9–98.1/ 98.7–99	96.0–96.7/ 96.8–97.8	99.6–100/ 100	98.2–99.1/ 98.6–99.1	98.2–98.9/ 98.9	97.6–99.1/ 98.8–99.7	97.5–99.2/ 93.5–97.5

**Table 4:** Clinical observations of challenged piglets.

DPI	Lethargy and Anorexia	Fecal Consistency		
		Normal	Mild Diarrhea	Watery Diarrhea
1	3/5	4/5	1/5	0/5
2	5/5	2/5	2/5	1/5
3	5/5	1/5	2/5	2/5
4	5/5	0/5	3/5	2/5
5	5/5	0/5	1/5	4/5
6	2/2	0/2	0/2	2/2
7	2/2	0/2	0/2	2/2
8	2/2	0/2	0/2	2/2
